# Progressive Multifocal Leukoencephalopathy in a Young Medication-Noncompliant HIV Patient: A Case Report and Literature Review

**DOI:** 10.7759/cureus.76747

**Published:** 2025-01-01

**Authors:** Nyan Lin Aung, Ruth Mamo, Gianna Castellano, Abrisham Akbariansaravi

**Affiliations:** 1 Internal Medicine, Richmond University Medical Center, Staten Island, USA; 2 Internal Medicine, American University of Antigua, Osbourn, ATG

**Keywords:** immune reconstitution syndrome, immunocompromised patients, medication noncompliance, progressive multifocal leukoencephalopathy (pml), young adult female

## Abstract

Progressive multifocal leukoencephalopathy (PML) is a rare and debilitating opportunistic infection of the central nervous system (CNS) caused by the John Cunningham (JC) virus. It predominantly affects immunocompromised individuals, including those with advanced HIV infection. The incidence of PML in people living with human immunodeficiency virus (HIV) has significantly declined in the era of antiretroviral therapy (ART). We report a case of PML in a young patient with a history of medication non-compliance, presenting with progressive neurological deficits, underscoring the importance of adherence to ART in preventing opportunistic infections.

## Introduction

Progressive multifocal leukoencephalopathy (PML) is a severe demyelinating disease caused by the reactivation of the John Cunningham (JC) virus, predominantly affecting immunocompromised individuals, particularly those with advanced human immunodeficiency virus (HIV) infection. The JC virus is a common virus that typically remains dormant in most individuals, but can reactivate in people with weakened immune systems, particularly those with advanced HIV infection. PML is a rare but often fatal brain infection that results from the reactivation of the JC virus. It causes damage to the white matter of the brain, leading to neurological deficits such as weakness, vision problems, and speech difficulties. In individuals living with HIV, antiretroviral therapy (ART) is critical because it strengthens the immune system by reducing the amount of virus in the body, thus preventing opportunistic infections like PML. This report presents the case of a 25-year-old female patient with a history of vertically transmitted HIV, who presented with acute neurological symptoms that were ultimately diagnosed as PML. Her clinical presentation underscores the need for early recognition and management of PML in patients with HIV, highlighting the importance of adherence to ART to prevent such life-threatening complications.

## Case presentation

**Figure 1:**
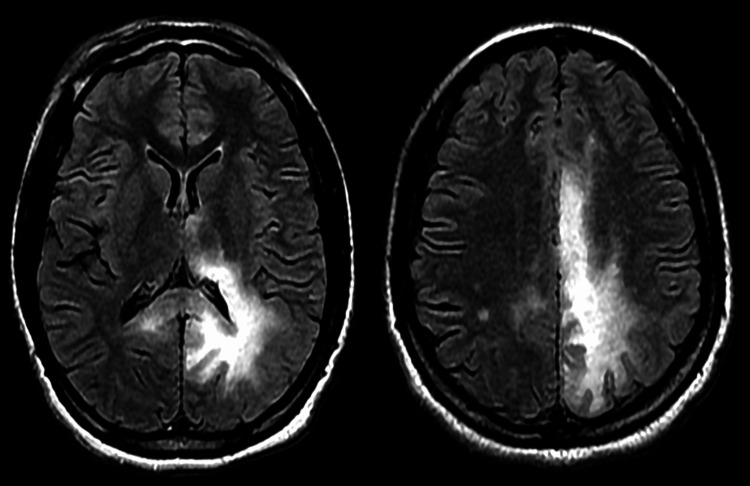
MRI of diffuse T2 FLAIR showed hyperintensity involving the left corona radiata, parietal, occipital region, and thalamus/basal ganglia FLAIR: fluid-attenuated inversion recovery

This is a case of a 25-year-old female with a past medical history of a human immunodeficiency virus (HIV) infection transmitted from her mother at birth; she was also non-compliant with her medications. Three months prior to admission, she was on combination ART with Biktarvy (bictegravir/emtricitabine/tenofovir 50-200-25 mg) and received prophylactic treatment with azithromycin and atovaquone due to profound immunosuppression, reflected by a low CD4 count of 56 cells/μL and a high viral load of 25,000 copies/mL. However, she is not compliant with her medication, leading to her presentation with progressive weakness on the right side of her body for two days, and had experienced intermittent headaches for a week. Upon neurological examination, the patient exhibited right-sided weakness (graded 4/5 strength), numbness, hyperreflexia (increased reflexes), and a positive Babinski sign on the right side. Additionally, she had an unsteady gait, suggesting focal neurological deficits. The patient was then admitted for right-sided hemiplegia. During the hospital stay, a brain magnetic resonance imaging (MRI) scan with/without contrast was performed and found diffuse T2 fluid-attenuated inversion recovery (FLAIR) hyperintensity involving the left corona radiata, parietal, occipital region, thalamus/basal ganglia with involvement of the subcortical U fibers with signal intensities (Figure 1).

MRI findings of non-enhancing T2 FLAIR hyperintensity involving the left corona radiata, parietal, and occipital regions are characteristic of PML. The involvement of subcortical U fibers, which are often affected in PML, correlates with the patient’s neurological deficits, including right-sided hemiplegia and gait disturbances. These findings help distinguish PML from other conditions such as HIV encephalopathy or posterior reversible encephalopathy syndrome (PRES), which can present with different imaging features. The laboratory tests showed normal hemogram and biochemistry but a low CD4 count of 70 cells/µL and viral load of 27 copies/mL. A CD4 count of 70 cells/µL places the patient in the category of severe immunosuppression, making her highly vulnerable to opportunistic infections like PML. Although the viral load is relatively low (27 copies/mL), suggesting some degree of viral control, the prolonged ART non-compliance likely led to chronic immunodeficiency and the reactivation of the JC virus. The cerebrospinal fluid examination confirmed the diagnosis of PML by detecting a JC virus polymerase chain reaction (PCR); it also showed elevated total protein (64 mg/dl) with 0 (cells/mm^3^) of WBCs and RBCs. The elevated protein levels in the CSF without the presence of WBCs or RBCs are consistent with the typical findings in PML, where inflammation is often absent. The positive JC virus PCR further supports the diagnosis. During her hospital stay, the patient's right-sided weakness worsened (3/5 strength) notably, raising suspicion of immune reconstitution inflammatory syndrome (IRIS) associated with her HIV treatment. PML-IRIS occurs when the immune system, reconstituted by ART, mounts an inflammatory response to the JC virus, potentially worsening neurological symptoms. This patient’s progressive right-sided weakness, despite treatment, raises the suspicion of PML-IRIS. Treatment with high-dose methylprednisolone was initiated to control the inflammatory response. While steroids can help reduce IRIS-related inflammation, the clinical outcomes are often unpredictable, and patients may continue to experience neurological decline despite treatment. However, our patient showed stable clinical condition and she was discharged to an acute short-term rehabilitation center. She was provided health education on the importance of medication compliance along with prophylaxis for opportunistic infections.

## Discussion

Pathophysiology and clinical significance

PML is a demyelinating disease caused by the reactivation of the JC virus in immunocompromised patients. This virus, which typically remains dormant, reactivates in those with weakened immune systems, especially patients with HIV who are non-adherent to ART. In this patient, the CD4+ count had fallen to 70 cells/µL, and the viral load was 27 copies/mL due to long-term non-compliance with ART, allowing the virus to reactivate and damage the brain’s oligodendrocytes, leading to severe neurological impairment [1,2].

JC virus reactivation is strongly linked to severe immunosuppression. When ART is not adhered to, as in this case, the immune system becomes incapable of controlling latent viral infections, resulting in conditions such as PML. The patient’s vertical transmission of HIV presents additional challenges, as it places the individual at a lifelong risk of immunosuppression, especially if ART adherence is inconsistent. Studies show that individuals with vertically transmitted HIV, like this patient, are particularly vulnerable to opportunistic infections due to the difficulty in maintaining lifelong treatment adherence [3].

PML can present with weakness, speech disturbances, cognitive impairment, headache, gait abnormality, seizures, sensory loss, and visual impairments. PML occurs most commonly in the periventricular and subcortical white matter in the parieto-occipital or frontal lobes. Although rare, lesions in the brainstem, cerebellum, and spinal cord have previously been described [4]. PML is typically diagnosed through MRI, which reveals characteristic non-enhancing hyperintense white matter lesions. In this patient, such lesions were confirmed through imaging, and a positive PCR test for JC virus DNA in CSF confirmed the diagnosis. While ART can help restore immune function and control viral replication, its re-initiation carries a risk of IRIS, where the immune system’s recovery paradoxically causes inflammation and worsens neurological outcomes. This complication must be considered in patients with advanced disease like this one [5].

Literature review

Several studies have explored PML in the context of vertically transmitted HIV. Adolescents and young adults with vertically transmitted HIV are at increased risk of non-compliance with ART, leading to prolonged periods of immunosuppression and an elevated risk of PML. In cases like this one, the prolonged non-adherence allowed JC virus reactivation, as also seen in similar case reports [6,7].

A recent study highlighted the poor prognosis associated with PML in HIV patients who present with advanced immunosuppression, similar to this patient. The neurological damage caused by the demyelinating effects of the JC virus is often irreversible, particularly in patients with delayed ART re-initiation [8]. Treatment outcomes vary but even aggressive management strategies, such as the use of mefloquine or mirtazapine in combination with ART, have shown limited success in reversing advanced PML [9,10]. The prognosis remains guarded, with high mortality rates reported in patients with advanced disease at the time of diagnosis.

This case highlights the difficulty in early diagnosis of PML, particularly in patients with long-standing non-compliance to ART. By the time PML is suspected, patients often present with significant neurological deficits, complicating the treatment process. About 55-80% of ΡML survivors are left with severe neurologic sequelae [11]. Studies suggest that consistent ART adherence from an early stage can significantly reduce the risk of JC virus reactivation and the development of PML [12,13].

Clinical implications

The central lesson from this case is the critical importance of maintaining consistent ART adherence in patients with vertically transmitted HIV. Prolonged noncompliance leads to severe immunosuppression, increasing the risk of life-threatening opportunistic infections like PML. Clinicians must closely monitor adherence in these patients, particularly during adolescence, when the risk of non-compliance increases. Early interventions, including education on the importance of adherence and regular viral load and CD4+ count assessments, are essential to prevent such severe complications [14].

This case further underscores the need for a multidisciplinary approach to managing HIV-positive patients. Psychosocial support, patient education, and adherence counseling should be integrated into routine care to ensure long-term ART adherence. By providing such support, clinicians can help prevent the development of catastrophic opportunistic infections like PML, which have a poor prognosis once neurological damage has occurred [11].

## Conclusions

This case illustrates the severe consequences of advanced HIV infection and poor treatment adherence, leading to profound immunosuppression and the development of progressive multifocal leukoencephalopathy (PML). Early recognition of neurological symptoms and appropriate diagnostic evaluation, including neuroimaging and cerebrospinal fluid analysis, are crucial for identifying PML and initiating supportive care. It emphasizes the importance of strict adherence to antiretroviral therapy (ART), to minimize the risk of irreversible neurological deficits, particularly in young adults. It also reinforces the need for ongoing patient education and multidisciplinary support to improve treatment adherence and long-term outcomes in vulnerable populations.
